# Theoretically Guided Analytical Method Development and Validation for the Estimation of Rifampicin in a Mixture of Isoniazid and Pyrazinamide by UV Spectrophotometer

**DOI:** 10.3389/fchem.2017.00027

**Published:** 2017-04-28

**Authors:** Mohammad F. Khan, Shamima A. Rita, Md. Shahidulla Kayser, Md. Shariful Islam, Sharmeen Asad, Ridwan Bin Rashid, Md. Abdul Bari, Muhammed M. Rahman, D. A. Anwar Al Aman, Nurul I. Setu, Rebecca Banoo, Mohammad A. Rashid

**Affiliations:** ^1^Department of Pharmacy, State University of BangladeshDhaka, Bangladesh; ^2^Department of Pharmaceutical Chemistry, University of DhakaDhaka, Bangladesh

**Keywords:** computational study, physicochemical properties, solubility, distribution coefficient, extraction, analytical method development, validation

## Abstract

A simple, rapid, economic, accurate, and precise method for the estimation of rifampicin in a mixture of isoniazid and pyrazinamide by UV spectrophotometeric technique (guided by the theoretical investigation of physicochemical properties) was developed and validated. Theoretical investigations revealed that isoniazid and pyrazinamide both were freely soluble in water and slightly soluble in ethyl acetate whereas rifampicin was practically insoluble in water but freely soluble in ethyl acetate. This indicates that ethyl acetate is an effective solvent for the extraction of rifampicin from a water mixture of isoniazid and pyrazinamide. Computational study indicated that pH range of 6.0–8.0 would favor the extraction of rifampicin. Rifampicin is separated from isoniazid and pyrazinamide at pH 7.4 ± 0.1 by extracting with ethyl acetate. The ethyl acetate was then analyzed at λ_max_ of 344.0 nm. The developed method was validated for linearity, accuracy and precision according to ICH guidelines. The proposed method exhibited good linearity over the concentration range of 2.5–35.0 μg/mL. The intraday and inter-day precision in terms of % RSD ranged from 1.09 to 1.70% and 1.63 to 2.99%, respectively. The accuracy (in terms of recovery) of the method varied from of 96.7 ± 0.9 to 101.1 ± 0.4%. The LOD and LOQ were found to be 0.83 and 2.52 μg/mL, respectively. In addition, the developed method was successfully applied to determine rifampicin combination (isoniazid and pyrazinamide) brands available in Bangladesh.

## Introduction

**Figure 1:**
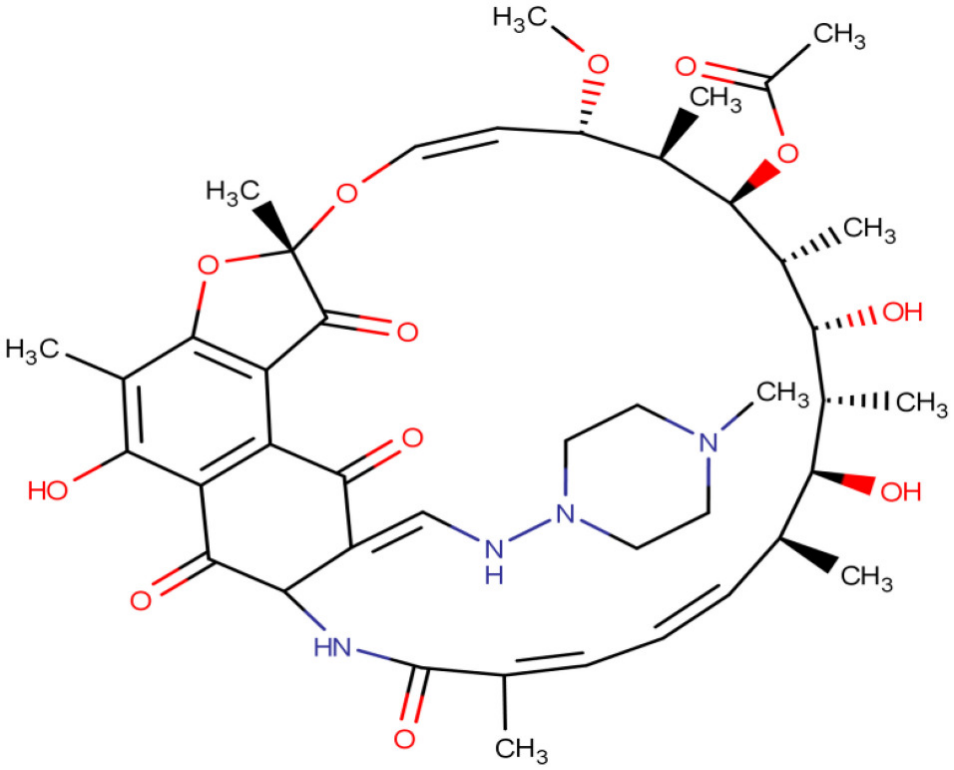
**Two dimensional structure of rifampicin**.

Rifampicin (Figure 1) is a semisynthetic antibiotic produced from *Streptomyces mediterranei*. It has a broad antibacterial spectrum, including activity against several forms of Mycobacterium (Brunton et al., 1996). In susceptible organisms, it inhibits DNA-dependent RNA polymerase activity by forming a stable complex with the enzyme and hence it suppresses the initiation of RNA synthesis (Desai and Shah, 2015). Rifampicin is bactericidal and acts on both intracellular and extracellular organisms (Brunton et al., 1996). It is used as the first line therapy in the treatment of tuberculosis (Desai and Shah, 2015).

Literature survey revealed numerous analytical techniques including HPLC (Smith et al., 1999; Calleja et al., 2004; Kumar et al., 2004; Tatarczak et al., 2005; Ali et al., 2007; Allanson et al., 2007; Hartkoorn et al., 2007; Liu et al., 2008; Bhusari et al., 2009; Sabitha et al., 2009; Wang et al., 2012; Mansuri et al., 2014; Moreno-Exebio and Grande-Ortiz, 2014) and UV spectroscopic methods (Rote and Sharma, 1997; Benetton et al., 1998; Khamar and Patel, 2012a,b; Tella et al., 2012; Begum et al., 2013; Priyanshu and Madhav, 2013) are available to assay rifampicin alone and in combination with other agents. Although the chromatographic methods are extensively used and recommended, but these methods require complex and expensive instrumentation, provision for use and disposal of solvents, labor-intensive sample preparation procedures and personal skills. On the other hand, simple spectrophotometric methods are available to assay rifampicin alone (Tella et al., 2012; Priyanshu and Madhav, 2013) but in case of combine dosage form the reported assay methods are quite complex (Khamar and Patel, 2012b). Since simple UV spectrophometric method for the assay of rifampicin in combined dosage form is not available, the goal of the present investigation was to develop and validate a simple, rapid, reliable and economical UV spectrometric method (guided by theoretical study) for the analysis of rifampicin in combined dosage form. Theoretical study was conducted to calculate physicochemical properties such as acid dissociation constant (pKa), partition coefficient (logP), distribution coefficient (logD), solubility (logS) in water and in different organic solvents of isoniazid, pyrazinamide and rifampicin. These properties guided the selection of suitable solvent for extraction and pH of the solution. However, the developed method was applied successfully for the estimation of rifampicin in presence of isoniazid and pyrazinamide.

## Materials and methods

### Theoretical investigations

The acid dissociation constant (pKa), partition coefficient (logP), distribution coefficient (logD), aqueous solubility (logS), and isoelectric point (pI) of isoniazid, pyrazinamide and rifampicin over the pH range of 0.0–14.0 at 298 K were calculated using MarvinSketch [Marvin 15.06.29, 2015, ChemAxon (http://www.chemaxon.com)].The consensus method was applied to calculate partition and distribution coefficient of the three molecules. Moreover, the solubility of these compounds in 80 different solvents was also calculated using Abraham descriptors (Abraham et al., 2010) and with the help of online software (http://showme.physics.drexel.edu/onsc/models/AbrahamDescriptorsModel001.php). However, we also calculate the enthalpy, Gibbs free energy and entropy of isoniazid, pyrazinamide and rifampicin in gas phase, water and ethyl acetate with the Gaussian 09 software package (Frisch et al., 2009). The geometries were optimized at the semi-empirical/AM1 (Austin Model 1) level of theory before performing any calculation. Frequencies were calculated to ensure the absence of imaginary frequencies in the lowest energy state. The Solvation Model on Density (SMD) (Marenich et al., 2009) as implemented in Gaussian 09 was used for all calculations involving the solvents.

### Experimental investigations

#### Drugs and reagents

Rifampicin (99.6%) was kindly gifted by Novartis (Bangladesh) Limited, Bangladesh. Ethyl acetate was of analytical grade and used without further purification.

#### Instrumentation

A UV spectrophotometer (UV- 1800 240V, SHIMADZU) equipped with a desktop (Intel® Core™ i3 processor) having UVprobe multifunctional UV control software was used to develop and validate the suggested method.

A simple UV spectroscopic method was developed and validated for the analysis of rifampicin in a combined solid dosage form. The development and validation procedure is as follows.

#### Preparation of standard solution (stock solution)

Ten (10) mg of standard rifampicin was measured using electronic balance (Model-ATY224, SHIMADZU) and diluted to 100 ml with distilled water to prepare a standard solution of concentration of 100 μg/mL.

#### Preparation of blank

Ten (10) mL of ethyl acetate and 10 mL of water was taken in a separating funnel and shaken well. The ethyl acetate layer was then collected in a volumetric flask. This solution was used as blank.

#### Determination of λ_max_

Two (2.0) mL of stock solution was diluted to 10 mL using distilled water. This solution was then extracted by 10 mL of ethyl acetate. The diluted ethyl acetate solution was scanned from 200 to 400 nm in UV spectrophotometer to determine the λ_max_.

#### Extraction ratio

The water and ethyl acetate were mixed at a ratio of (water: ethyl acetate) 1: 0.25, 1: 0.5, 1: 1, 1: 1.5, and 1: 2 to get the optimum extraction ratio.

#### Extraction time

The mixtures (water: ethyl acetate = 1: 1 (v/v)) were allowed to stand for different time interval such as 1.0, 1.5, 2.0, and 3.0 h to find the best separation time.

#### Analytical method validation

The ICH guidelines (International Conference on Harmonisation (ICH), 2005) were followed to validate the analytical method. The newly developed UV spectrophotometric method was validated for parameters like specificity, linearity, accuracy, precision, limit of detection (LOD) and limit of quantitation (LOQ).

#### Specificity test

The specificity of the method was confirmed through establishing the identity of rifampicin by Thin Layer Chromatographic (TLC) technique. The standard rifampicin solution and the extracted sample (tablet) solution were spotted on Thin Layer Chromatography (TLC) plates (Silica gel F_254_) and the plates were developed using mobile phase comprising of 10 and 15% methanol in chloroform.

#### Linearity and range

The linearity graphs were plotted for the concentration range of 2.5–35.0 μg/ml. Least square analysis was used to obtain the slope, intercept, correlation coefficient (*R*^2^) and regression equation of the method.

#### Accuracy

Accuracy represents the bias of the method. Five different concentrations such as 10 μg/mL, 15 μg/mL, 20 μg/mL, 25 μg/mL, 30 μg/mL, and 35 μg/mL of standard rifampicin solution were used to find the accuracy of the method. Three replicates of each concentration were used and then their average concentration was used to calculate the accuracy by applying the following equation:

$$\begin{array}{l} \text{Accuracy }\left(\%\right)=\;\frac{\text{Observed concentration}}{\text{Actual concentration}}\times 100 \end{array}$$

The accuracy should be within 15% of the actual value, except at the lower limit of quantitation (LOQ), where it should be ≤20% (Xiong et al., 2009).

#### Precision

Precision of the assay was assessed with respect to repeatability (intraday) and intermediate precision (inter-day). The precision of the current method was determined by analyzing three different concentrations. Three replicates of each concentration were used to calculate the relative standard deviation (RSD) by following the equation below:

$$\begin{array}{l} \text{RSD }\left(\%\right)=\;\frac{\text{Standard deviation}}{\text{Mean}}\times 100 \end{array}$$

The calculated RSD at each concentration level should not exceed 15%, except for LOQ, where it should not exceed 20% (Mostafavi et al., 2009).

#### Limit of detection (LOD)

The LOD was calculated according to following equation:

$$\begin{array}{l} \text{L}OD=\;\frac{3.3\times SD}{\sigma } \end{array}$$

Where, SD = standard deviation of response

σ = slope of regression line

#### Limit of quantification (LOQ)

The LOQ is the measure of an analytical method that can quantify an analyte with adequate accuracy and precision and it can be calculated as follows:

$$\begin{array}{l} \text{L}OQ=\;\frac{10\times SD}{\sigma } \end{array}$$

Where, SD = standard deviation of response

σ = slope of regression line

#### Application of the method

Two brands of combined solid dosage form of rifampicin, isoniazid and pyrazinamide are available in Bangladesh. So, the two brands (coded S1 and S2) were assayed. The powdered form of each drug (equivalent to 10 mg of rifampicin) was taken in a 100 mL volumetric flask and dissolved in distilled water. Sonication was done for about 20 min and the solution was then diluted up to the mark with distilled water. The solution was then filtered through Whatman No. 1 filter paper. The filtered solution was further diluted according to the need and extracted by using 10 ml of ethylacetate. Ethyl acetate layer was collected in a volumetric flask. Absorbances of these ethyl acetate solutions were determined.

#### Comparison with the reported method

The newly developed method was compared with the reported Q-absorbance ratio spectrophotometric method (Khamar and Patel, 2012b). The two methods are employed to assay rifampicin following the method described above and the difference between the results of two methods was statistically evaluated by applying *t*-test and ANOVA (single factor) analysis. The statistical calculations were performed using Microsoft® Excel 2007.

## Results and discussion

### Theoretical investigations

#### Calculation of acid dissociation constant (pKa)

**Figure 2:**
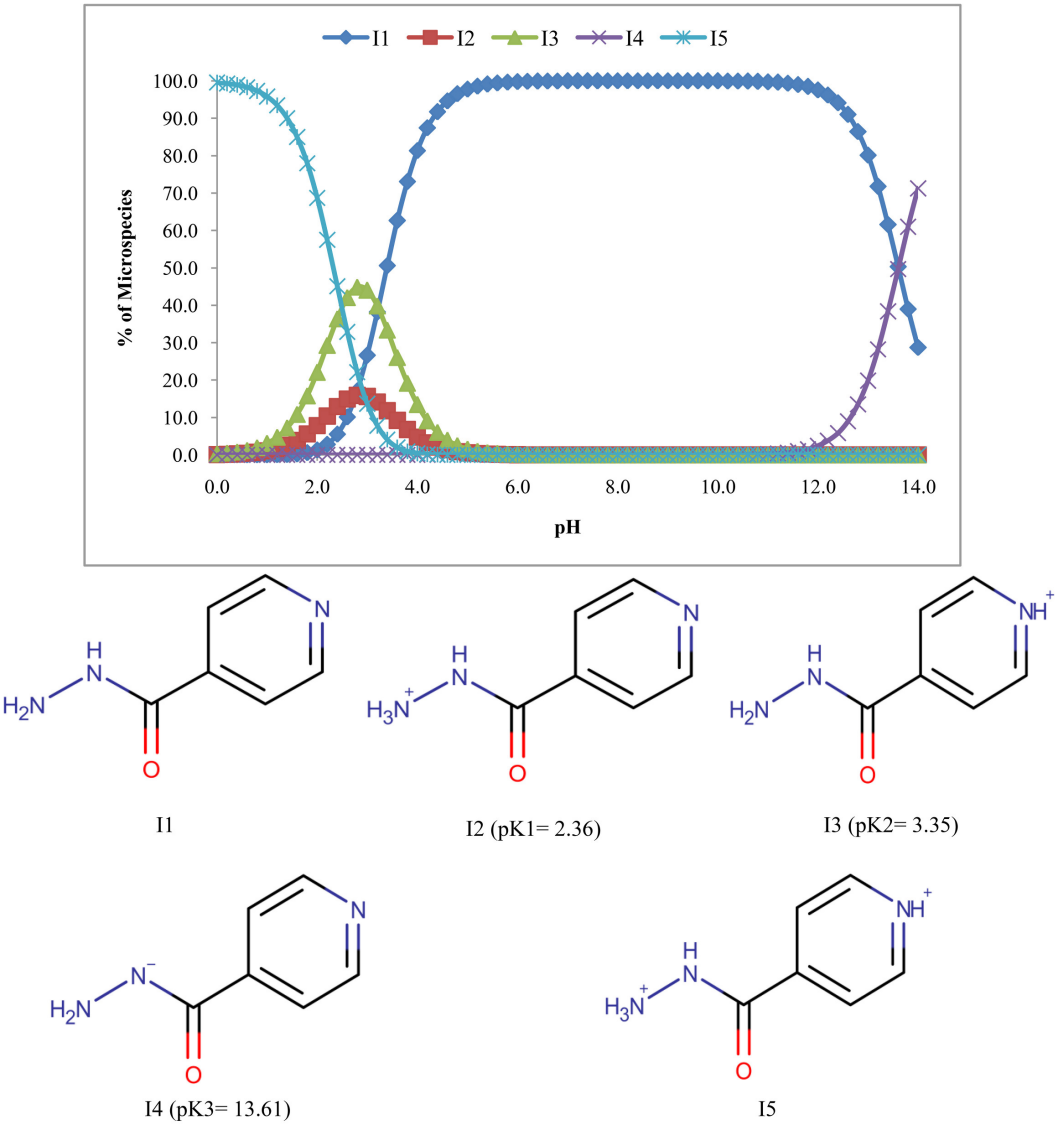
**Microspecies distribution (%) of isoniazid**.

Five microspecies with four ionized (I2, I3, I4, and I5) and one unionized form (I1) of isoniazid were found and only the unionized form (I1) predominates (100%) in the pH range of 6.8–10.2 (Figure 2).

**Figure 3:**
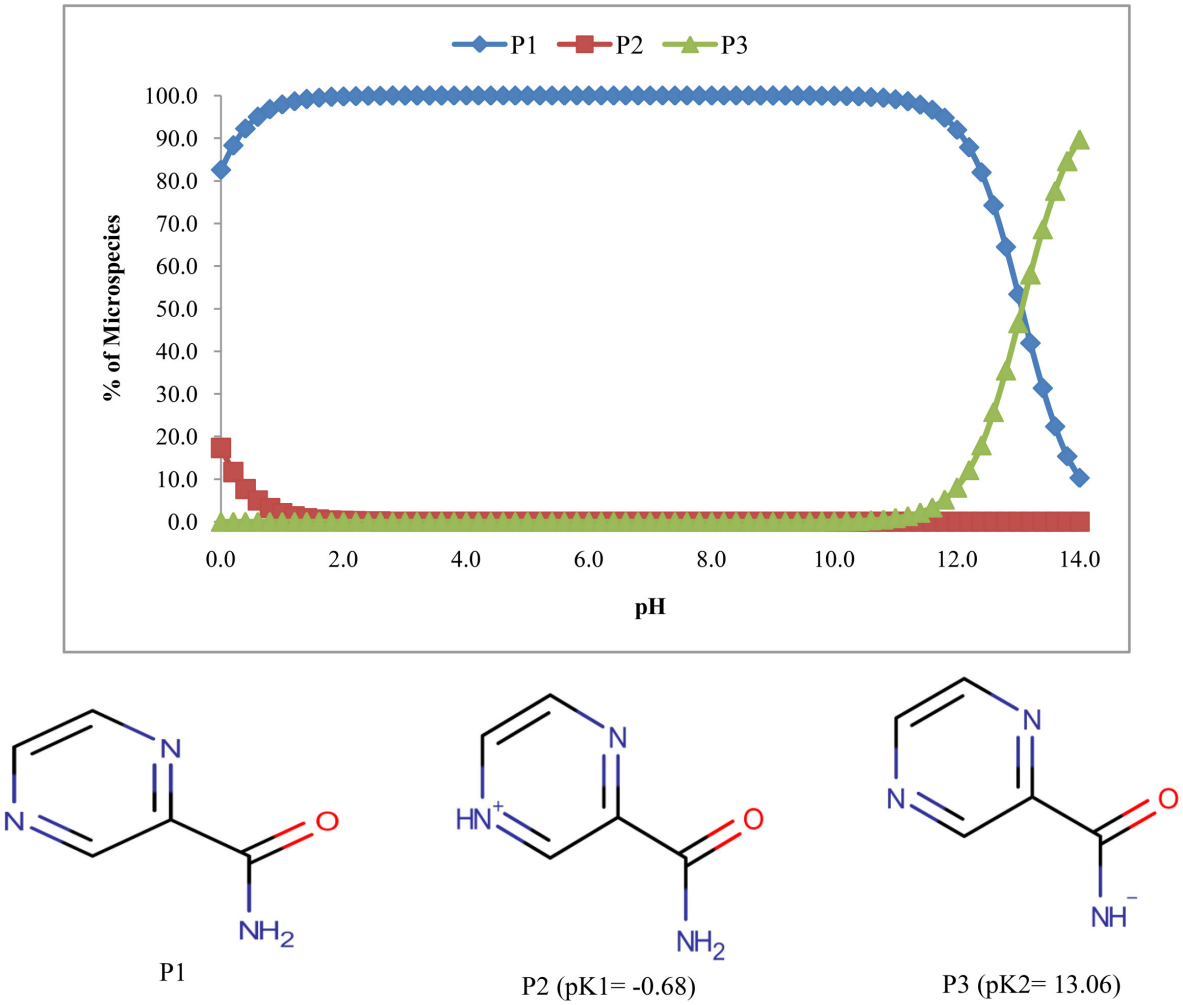
**Microspecies distribution (%) of pyrazinamide**.

The pyrazinamide exhibits three species one of which is unionized (P1) and the remaining two were ionized forms (P2 & P3) (Figure 3). The abundance of unionized form was 100% in the pH range of 2.6–9.6.

**Figure 4:**
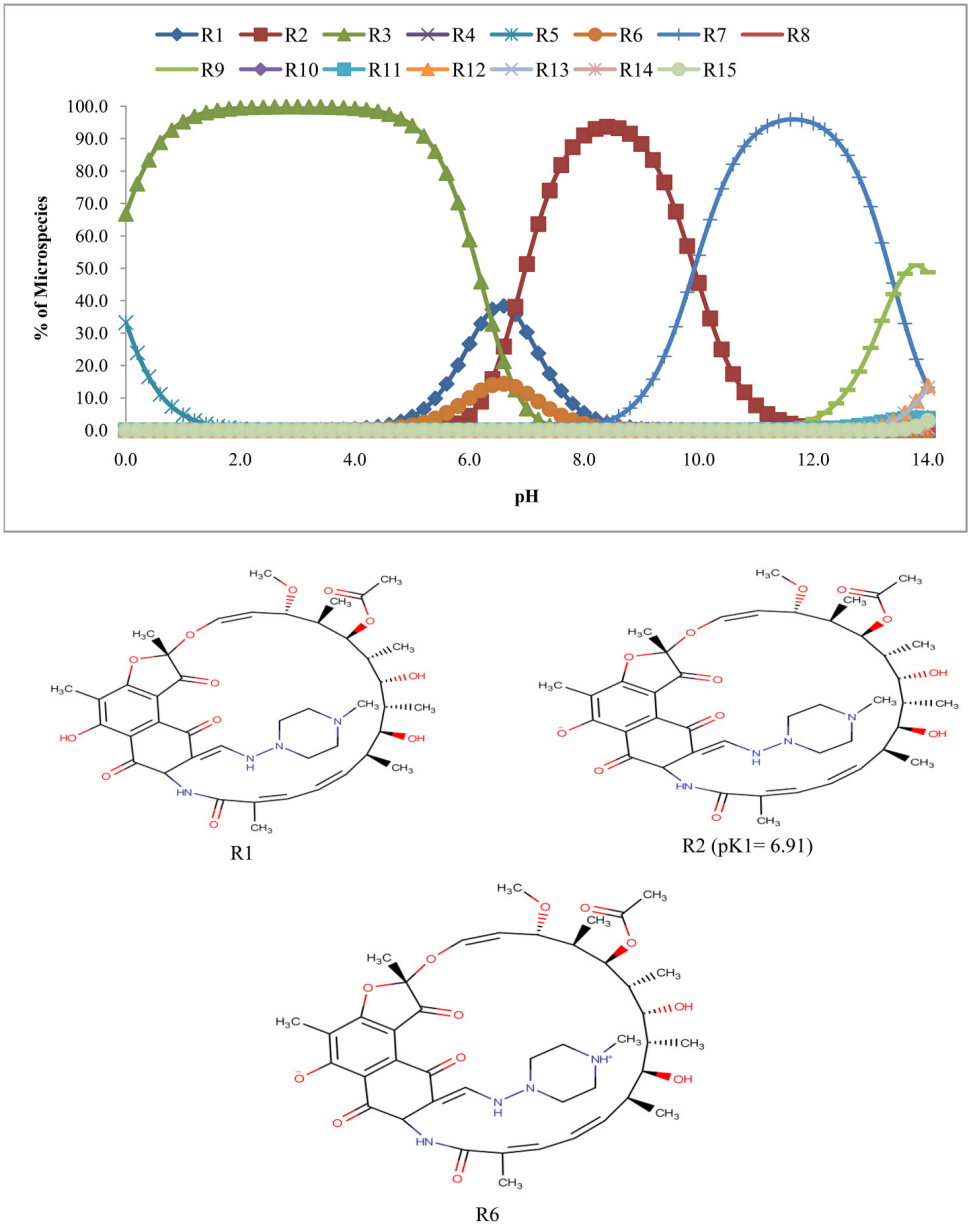
**Microspecies distribution (%) of rifampicin**.

The calculation of pKa over the pH of 0.0–14.0 revealed that rifampicin has 14 ionized species (R2–R15) along with the unionized form (R1) (Figure 4). It is clear from Figure 4 that from pH 6.8–7.6, three species of rifampicin (R1, R2, and R6) predominates (87.2–98.5% in abundance) in the solution.

#### Partition coefficient (logP)

The partition coefficient (logP) was calculated at 0.1M electrolyte concentration using consensus method. The logP value (Table 1) demonstrates that isoniazid and pyrazinamide are hydrophilic whereas rifampicin is lipophilic i.e., has higher affinity to *n*-octanol.

**Table 1:** **Partition coefficient of isoniazid, pyrazinamide and rifampicin**.

**Name of drug**	**logP**
Isoniazid	−0.69
Pyrazinamide	−1.23
Rifampicin	2.77

#### Distribution coefficient (logD)

**Figure 5:**
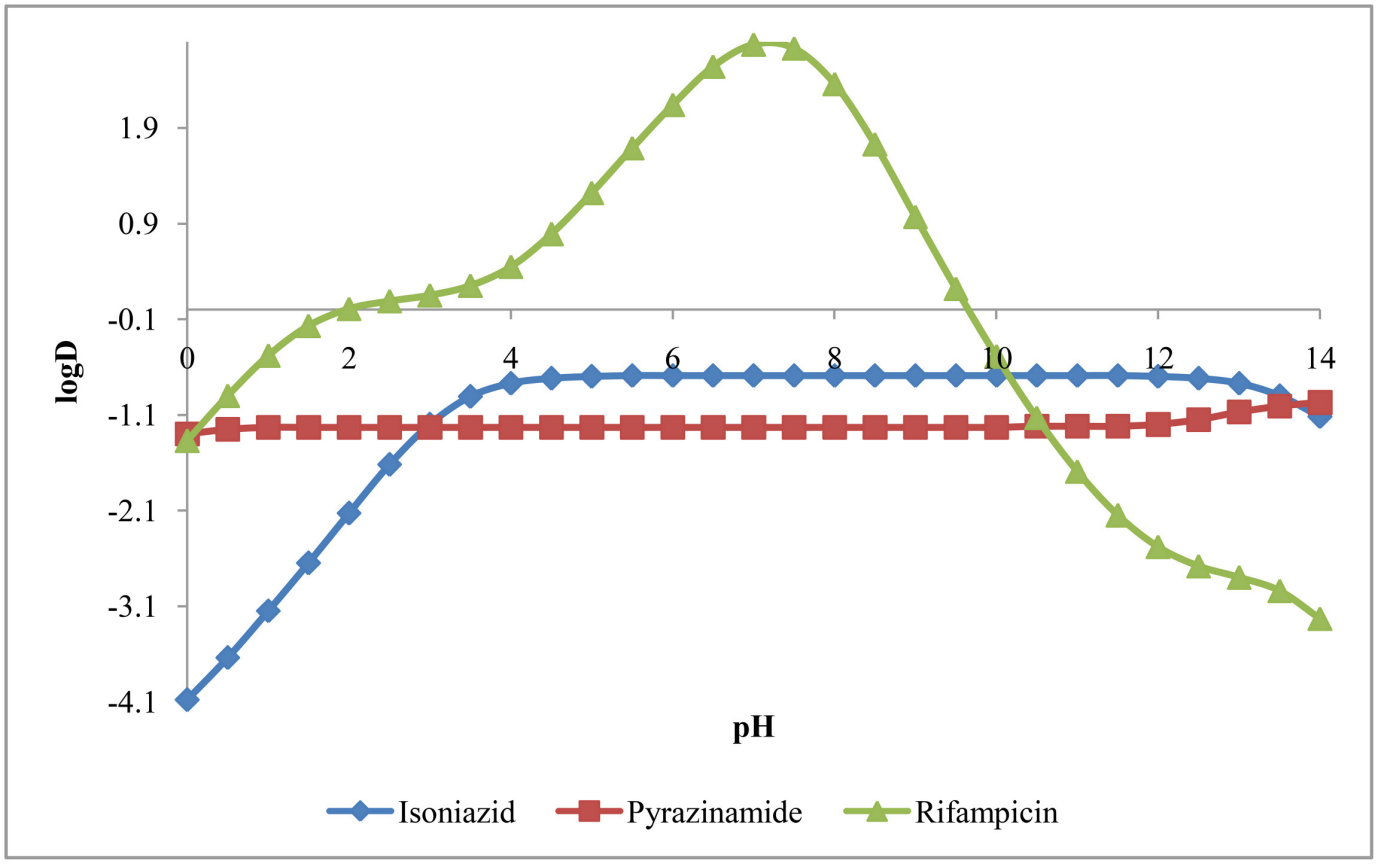
**Distribution coefficient (logD) of isoniazid, pyrazinamide and rifampicin**.

The logD was calculated at electrolyte concentration of 0.1M using consensus method implemented in MarvinSketch. The plot of logD vs. pH for isoniazid, pyrazinamide and rifampicin has been presented in Figure 5. From pH 0.0 to 14.0, the logD for isoniazid and pyrazinamide is negative in quantity, indicating both drugs have higher affinity and hence, higher solubility in water. However, rifampicin has logD value over 2 from pH 6.0 to 8.0 suggesting it has higher solubility in *n*-octanol over this pH range.

#### Aqueous solubility (logS)

**Figure 6:**
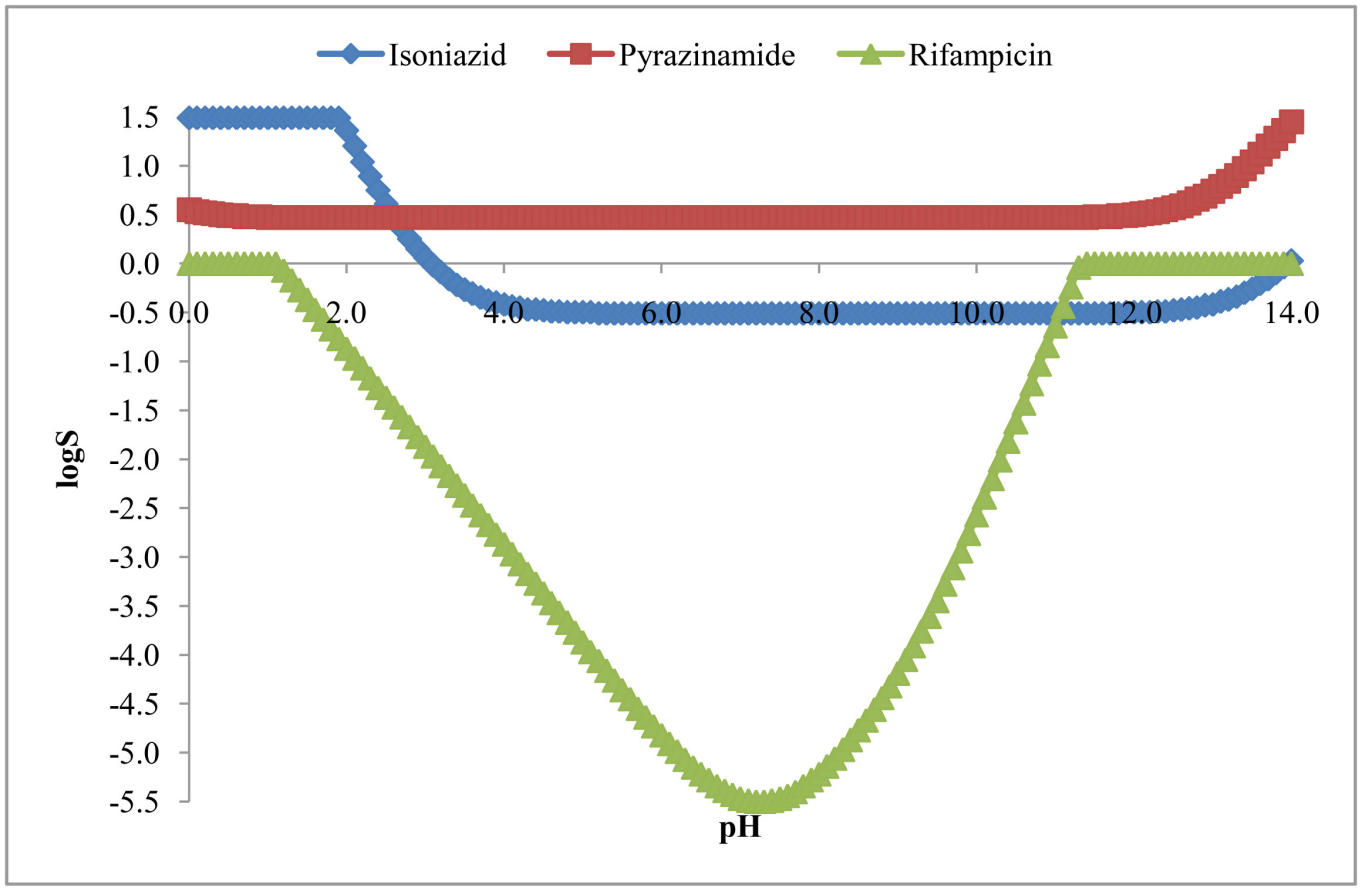
**Solubility (logS) of isoniazid, pyrazinamide and rifampicin**.

The aqueous solubility, in terms of logS, has been presented in Figure 6. The figure indicates that isoniazid, pyrazinamide and rifampicin over the pH of 5.2 to 10.8 were soluble (42.38 mg/mL), freely soluble (363.3 mg/mL) and practically insoluble (<0.1 mg/mL) in water, respectively. However, Table 2 represents intrinsic solubility of these drugs and suggests similar findings as described above.

**Table 2:** **Intrinsic solubility of isoniazid, pyrazinamide and rifampicin in water**.

**Name of drugs**	**Molecular weight**	**Intrinsic solubility in water**			**Remarks (Sinko, 2010)**
		**logS**	**mol/L**	**mg/mL**	
Isoniazid	137.14	−0.51	0.309	42.38	Soluble
Pyrazinamide	123.11	0.47	2.951	363.33	Freely soluble
Rifampicin	822.94	−6.4	0.0000004	0.0003	Practically insoluble

#### Isoelectric point (pI)

**Figure 7:**
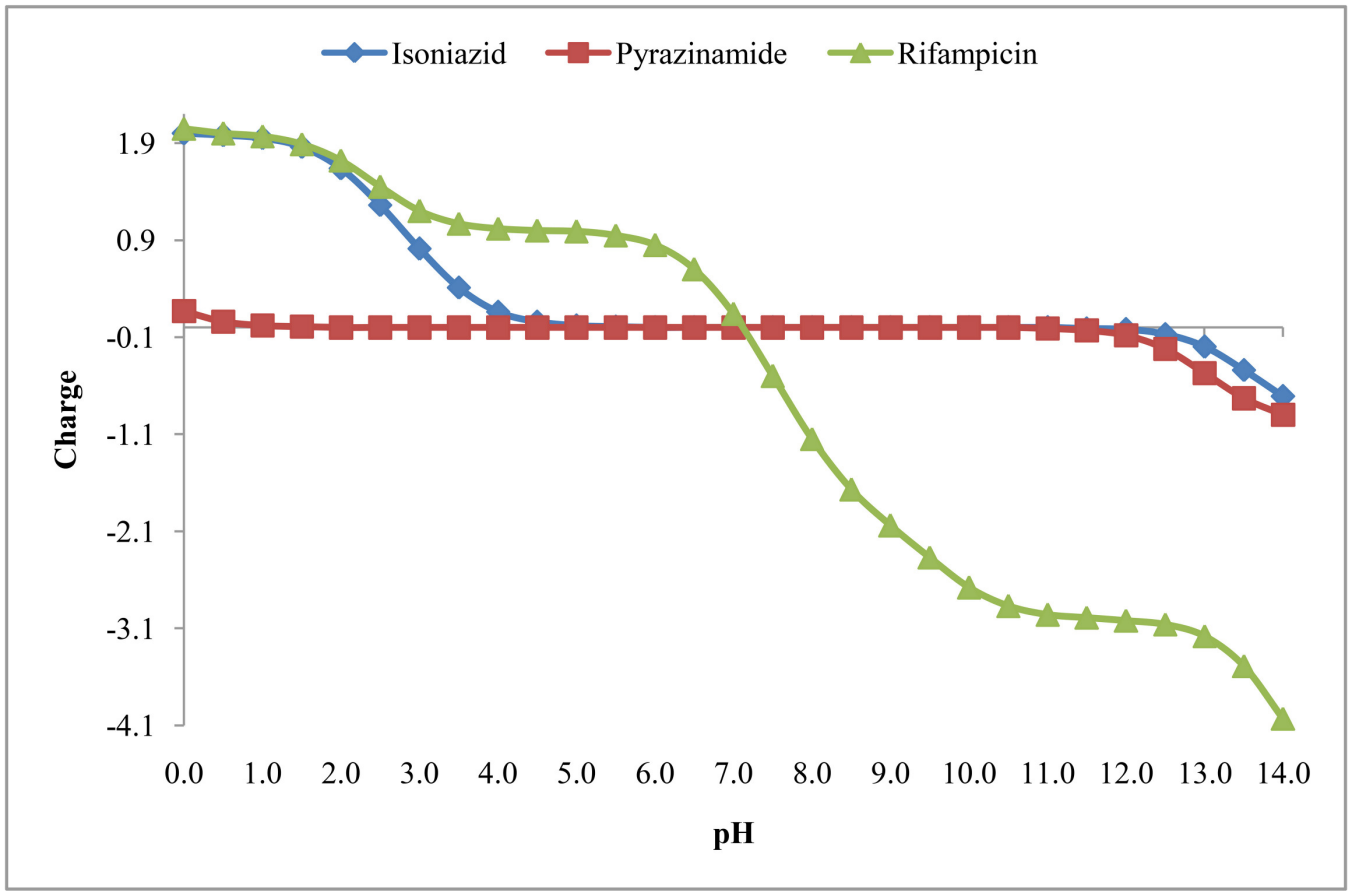
**Isoelectric point of isoniazid, pyrazinamide and rifampicin**.

The isoelectric point is the pH where the molecule has no net charge. The pI value of isoniazid, pyrazinamide and rifampicin is shown in Table 3 and Figure 7.

**Table 3:** **Isoelectric point of isoniazid, pyrazinamide and rifampicin**.

**Name of drugs**	**pI**
Isoniazid	8.45
Pyrazinamide	6.18
Rifampicin	7.11

Figure 4 suggests that rifampicin carries little or no net charges at pH near to 7.0 and hence, is suitable for extraction at this pH. Although isoniazid and pyrazinamide also has no net charge at this pH, but their higher solubility in aqueous media will retard their extraction.

#### Selection of solvent

The solubility of isoniazid, pyrazinamide and rifampicin were calculated in 80 different solvents using Abraham descriptors (Abraham et al., 2010). From the virtual screening it was found that 1,2-dichloroethane, 1-butanol, 1-decanol, 1-heptanol, 1-hexanol, 1-octanol, 1-pentanol, 2-methyl-2-propanol, butyl acetate, diethyl ether, ethyl acetate, fluorobenzene, isopropyl myristate, and octadecanol are suitable for the extraction of rifampicin from the water mixture. However, considering the availability, cost and hazard we choose ethyl acetate as an extracting solvent.

#### Solubility in ethyl acetate

The solubility of isoniazid, pyrazinamide and rifampicin were calculated in ethyl acetate using Abraham descriptors (Abraham et al., 2010). The calculated results (Table 4) revealed that isoniazid, pyrazinamide and rifampicin were slightly soluble (1.37 mg/mL), very slightly soluble (0.37 mg/mL) and freely soluble (107.81 mg/mL), respectively. Hence, ethyl acetate was chosen to extract rifampicin from the water mixture of isoniazid and pyrazinamide.

**Table 4:** **Isoelectric point of isoniazid, pyrazinamide and rifampicin**.

**Name of drugs**	**Molecular weight**	**Solubility in ethyl acetate**		**Remarks (Sinko, 2010)**
		**mol/L**	**mg/mL**	
Isoniazid	137.14	0.01	1.37	Slightly soluble
Pyrazinamide	123.11	0.003	0.37	Very slightly soluble
Rifampicin	822.94	0.131	107.81	Freely soluble

#### Selection of pH

Theoretical investigation of acid dissociation constant (pKa), partition coefficient (logP), distribution coefficient (logD), isoelectric point (pI), and solubility in water (logS) of isoniazid, pyrazinamide and rifampicin suggests that pH range of 6.0–8.0 will favor the extraction of rifampicin using organic solvent (ethyl acetate) as distribution coefficient is higher at this pH range. So, we extracted rifampicin using ethyl acetate at different pH such as 2.6, 4.1, 7.3, 8.3, and 9.7.

The results (Table 5) indicate that pH 7.3 would be suitable to extract rifampicin which is in accordance with our theoretical findings. So, the extraction of rifampicin was performed at pH 7.4 ± 0.1.

**Table 5:** **Effect of pH on extraction of rifampicin**.

**pH**	**Declared concentration (μg/ml)**	Average concentration found^*^ (μg/ml)
2.6	20.00	6.78 ± 0.02
4.1	20.10	18.86 ± 0.04
7.3	20.20	20.78 ± 0.05
8.3	20.00	15.91 ± 0.04
9.7	20.10	16.30 ± 0.07

* *Each value is the mean ± SD of three determinations*.

#### Calculation of enthalpy, gibbs free energy and entropy

The computed enthalpy, Gibbs free energy and entropy are presented in Table 6. The results suggest that rifampicin possesses higher entropy in ethyl acetate (−28.0 J/K/mol) as compared to water (−53.7 J/K/mol) whereas isoniazid and pyrazinamide have higher entropy in water (3.8 and −1.0 J/K/mol, respectively) than ethyl acetate (2.4 and −3.3 J/K/mol, respectively).

**Table 6:** **Change of enthalpy, Gibbs free energy and entropy of isoniazid, pyrazinamide and rifampicin relative to gas phase**.

**Name of drugs**	**Water**			**Ethyl acetate**			Δ **(Ethyl acetate–water)**		
	***ΔH (kJ/mol)***	***ΔG (kJ/mol)***	***ΔS (J/K/mol)***	***ΔH (kJ/mol)***	***ΔG (kJ/mol)***	***ΔS (J/K/mol)***	***ΔΔH (kJ/mol)***	***ΔΔG (kJ/mol)***	***ΔΔS (J/K/mol)***
Isoniazid	−30.7	−31.9	3.8	−11.6	−12.3	2.4	19.1	19.5	−1.5
Pyrazinamide	−49.0	−48.7	−1.0	−21.2	−20.2	−3.3	27.8	28.5	−2.4
Rifampicin	−173.9	−157.9	−53.7	−116.4	−108.0	−28.0	57.5	49.8	25.7

### Experimental investigation

#### Extraction ratio

The mixture of water and ethyl acetate at different ratio were investigated to get the optimum volume of water and ethyl acetate. Table 7 suggests that solvent ratio of (water: ethyl acetate) 1: 1, 1: 1.5, and 1: 2 are suitable to extract rifampicin from water. So, we select 1: 1 solvent ratio (lowest optimum volume of ethyl acetate) for extracting rifampicin.

**Table 7:** **Effect of extraction ratio of solvents**.

**Water : ethyl acetate**	**Declared concentration of rifampicin (μg/ml)**	Average concentration found^*^ (μg/ml)
1.0 : 0.25	20.00	15.16 ± 0.02
1.0 : 0.5	20.00	15.37 ± 1.38
1.0 : 1.0	20.00	20.2 ± 0.08
1.0 : 1.5	20.00	18.67 ± 0.45
1.0 : 2.0	20.00	19.8 ± 0.41

* *Each value is the mean ± SD of three determinations*.

#### Extraction time

The result of extraction is presented in Table 8. From the table it is clear that separation time for 2 h yielded good recovery with less variability. However, the room temperature was maintained to 25°C ± 0.5°C and the cap of the extracting apparatus were kept closed during the whole extraction time to minimize evaporation of ethyl acetate.

**Table 8:** **Separation time of the method**.

**Declared concentration (μg/ml)**	Average concentration found^*^ **(**μ**g/ml)**			
	**1.0 h**	**1.5 h**	**2.0 h**	**3.0 h**
10.00	10.99 ± 0.18	11.46 ± 0.11	11.14 ± 0.08	11.10 ± 0.06
20.00	21.26 ± 0.13	21.21 ± 0.15	20.85 ± 0.10	20.94 ± 0.15
30.00	31.01 ± 0.15	31.44 ± 0.04	30.23 ± 0.06	31.17 ± 0.11

* *Each value is the mean ± SD of three determinations*.

#### Specificity test

The specificity test was performed using Thin Layer Chromatographic (TLC) technique. The standard and extracted sample solution of rifampicin were spotted on Thin Layer Chromatography (TLC) plates (Silica gel F_254_) and the plates were developed using mobile phase consisting of 10 and 15% methanol in chloroform. A single brown spot was found for both solutions at R_f_ (Retardation factor) of approximately 0.54 when the developed plates were sprayed with vanillin/H_2_SO_4_ spray reagent.

#### Linearity and range

**Figure 8:**
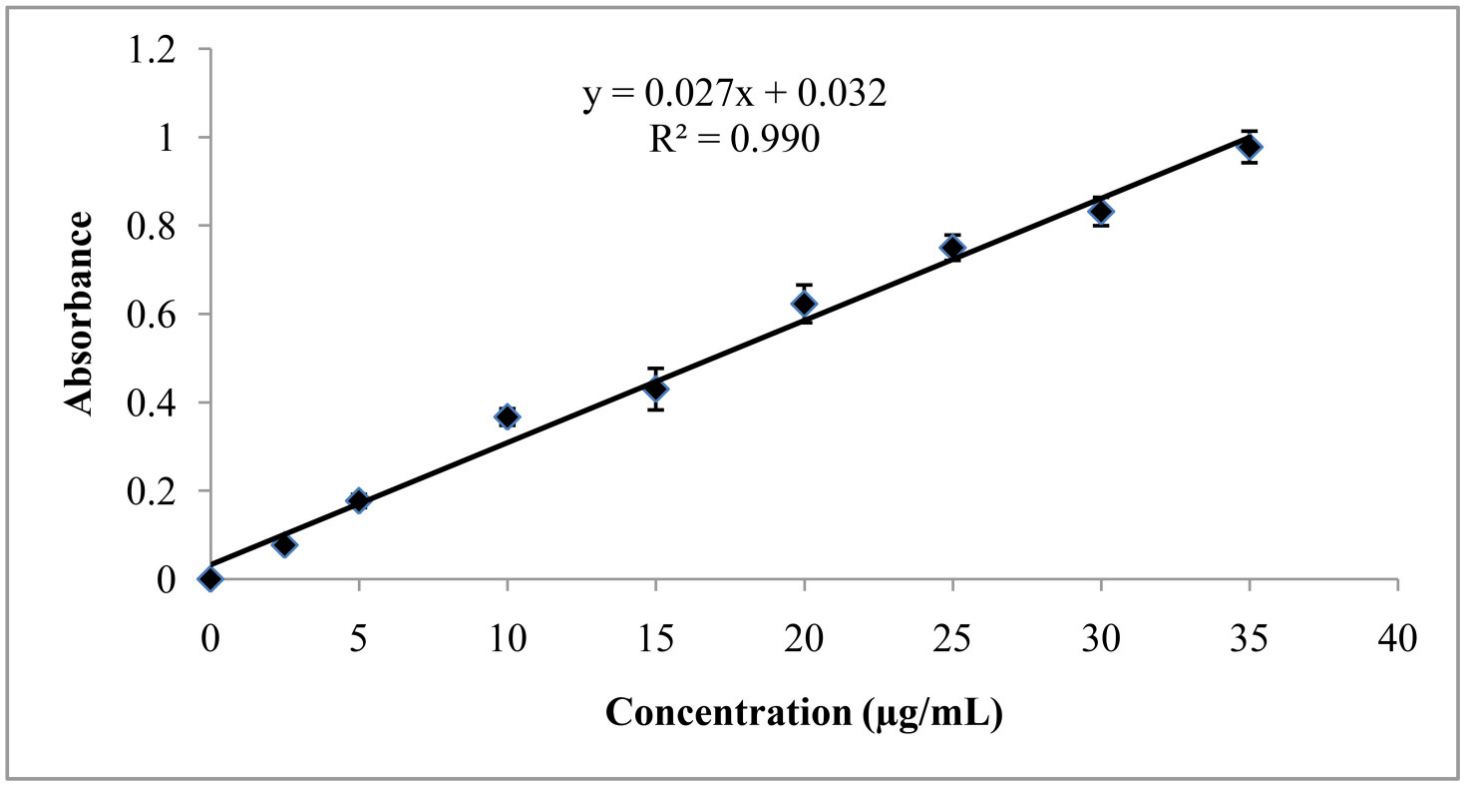
**Linearity study of rifampicin**.

The λ_max_ of rifampicin in ethyl acetate solution was found to be 344.0 nm. The absorbances of the sample solution were plotted against concentration range of 2.5–35.0 μg/mL and a good correlation coefficient was obtained. The correlation coefficient (*R*^2^) for the best fitting line was 0.990 which indicates good linear relationship of the newly developed method. The slope (m) and intercept (c) of the calibration curve were found to be 0.027 and 0.032, respectively (Figure 8 and Table 9).

**Table 9:** **Linearity and range of the method**.

**Parameter**	**Absorbance**
Linear concentration range (μg/ml)	2.5–35.0
Correlation coefficient (*R*^2^)	0.990
Slope (m)	0.027
Intercept (c)	0.032

#### Accuracy

The accuracy of the proposed method was in the range of 96.7 ± 0.9% to 101.1 ± 0.4% which is within the acceptable limit. The result of accuracy is presented in Table 10.

**Table 10:** **Accuracy of the method**.

**Declared concentration(μg/ml)**	Concentration found^*^ (μg/ml)	Accuracy^*^ (%)
10	11.06 ± 0.09	96.7 ± 0.9
15	15.98 ± 0.17	99.8 ± 1.1
20	20.80 ± 0.06	101.0 ± 0.3
25	25.51 ± 0.09	101.1 ± 0.4
30	30.02 ± 0.18	100.5 ± 0.6

* *Each value is the mean ± SD of three determinations*.

#### Precision

The precision of the method has been shown in Table 11. The intraday precision and inter-day precision in terms of % RSD was in the range of 1.09–1.70% and 1.63–2.99%, respectively.

**Table 11:** **Precision of the method**.

**Declared concentration (μg/ml)**	**Intra-day (*****n*** = **9)**		**Inter-day (*****n*** = **9)**	
	**Concentration found (μg/ml)**	**Precision (% RSD)**	**Concentration found (μg/ml)**	**Precision (% RSD)**
10.00	10.77	1.70	10.77	1.63
20.00	19.92	1.09	20.74	2.99
30.00	29.11	1.48	30.36	2.83

Since all the values of accuracy and % RSD of precision study were within the acceptable range, the results indicate that the method is reliable, reproducible and accurate.

#### Limit of detection and limit of quantification

The LOD and LOQ of the developed method were found 0.83 μg/mL and 2.52 μg/mL, respectively.

#### Application of the method

The assay result of the two marketed preparation S1 and S2, available in Bangladesh were 108.8 ± 1.21% and 115.5 ± 9.37%, respectively (Table 12).

**Table 12:** **Assay of marketed formulations**.

**Sample**	**Declared concentration (μg/ml)**	Average concentration found^*^ (μg/ml)	Recovery^*^ (%)	Potency^*^ (%)
S_1_	20.00	22.07 ± 0.24	110.4 ± 1.22	108.8 ± 1.21
S_2_		20.47 ± 1.66	102.3 ± 8.30	115.5 ± 9.37

* *Each value is the mean ± SD of nine determinations*.

#### Comparison with the reported method

The results of the proposed and the reported method (Khamar and Patel, 2012b) are presented in Table 13. At 95% confidence interval the calculated *F*_(6.323)_ and *t*_(2.515)_ value were less than their respective critical value (7.71 and 2.776, respectively). This indicates that the difference between the two methods is statistically insignificant. The comparison with the reported method shows that the developed method is accurate and precise.

**Table 13:** **Assay of marketed formulations**.

**Parameter**	**Rifampicin (*****n*** = **3)**	
	**Proposed method**	**Reported method (Khamar and Patel, 2012b)**
Label claim (mg)	300.0	
Amount found (mg)	310.5	307.5
% Amount found	103.5	102.5
*SD*	1.27	1.63
%RSD	1.23	1.60
*t* value	2.515	
*F* value	6.323	

## Conclusion

Rifampicin, a hydrophobic molecule (Williams and Piddock, 1998) is soluble in acidic and basic pH (Agrawal and Panchagnula, 2005). It has an aromatic ring system called naptokinone along with a long aliphatic bridge (Sköld, 2011) which might contribute to its hydrophobicity. Our theoretical investigation dictates that it has an isoelectric point at 7.11 and hence any form of significant solubility in water due to transient dipole moments is unlikely. Solubility of rifampicin in ethyl acetate is due to London forces since such forces are primarily prevalent in the interaction of hydrophobic molecules in non-polar solvents (London, 1937). It was seen that rifampicin has a lower ΔS value (lower entropy) (Table 6) in water than in ethyl acetate and hence it explains the lipophilicity/hydrophobicity of rifampicin. The results substantiate the claim that the solubility is dependent on the entropy of the solution (Baena et al., 2004). The findings of theoretical investigation indicated that selection of ethyl acetate would be suitable for extracting rifampicin from a water mixture of isoniazid and pyrazinamide at pH 7.4 ± 0.1.

The accuracy, intra-day and inter-day precision of the developed method were found in the range of 96.7 ± 0.9%–101.1 ± 0.4%, 1.09–1.70%, and 1.63–2.99%, respectively. The acceptable ranges for accuracy and precision are within 15% of the actual value (Xiong et al., 2009) and <15% (Mostafavi et al., 2009), respectively. In addition, the LOD and LOQ were 0.83 and 2.52 μg/ml, respectively.

Based on these investigations a simple, economic and effective UV spectroscopic method has been developed for the estimation of rifampicin present in a combined (isoniazid, pyrazinamide, and rifampicin) pharmaceutical dosage form. Previously, a simultaneous equation method was applied to assay a mixture of rifampacin and isoniazid by UV spectrophotometry (Begum et al., 2013). In that assay the linearity range, LOD, LOQ, mean accuracy, inter- and intra-day precision of rifampicin were 5–35 μg/mL, 1.653 μg/mL, 5.007 μg/mL, 99.23%, 0.784%, and 0.578%, respectively. However, ethanol, a costly solvent was used to perform the assay. Moreover, errors are common in simultaneous equation method and such errors can affect significantly the accuracy of results when spectra overlap significantly (Owen, 1996). Whether the spectra overlap was not mentioned in the assay developed by Begum et al. (2013). Our assay is free from such issues.

Khamar and Patel (2012b) developed a Q-absorbance ratio spectrophotometric method for the simultaneous estimation of rifampicin and piperine. The method was validated over the concentration range of 5–40 μg/mL with LOD, LOQ, accuracy, inter- and intra-day precision of rifampicin 0.8 μg/mL, 2.45 μg/mL, 99.24 ± 0.46%, 0.28–1.62%, and 0.17–1.31%, respectively. In that method, hazardous methanol (Hazardous Substance Fact Sheet)^1^ was used as a dissolving and diluting solvent.

The validation results of our proposed method indicate that the method is adequately linear over the specified concentration range (2.5–35.0 μg/mL, *R*^2^ = 0.990), accurate (96.7 ± 0.9%–101.1 ± 0.4%) and precise (intraday precision 1.09–1.70% and inter-day precision 1.63–2.99%). Moreover, the use of distilled water as a dissolving and diluting solvent warrants the cost effectiveness and safety of the suggested method. The successful application of the developed method proves its usefulness and workability. So, the developed method can be used for assaying combination products of rifampicin.

## Author contributions

Conceived and designed the experiments: MFK, RBR, and MSK. Performed theoretical investigations: MFK, MB, and MI. Performed the experiments: SR, MMR, and SA. Analyzed the data: MFK, DA, and NS. Wrote the paper: MFK, RB, and MAR.

### Conflict of interest statement

The authors declare that the research was conducted in the absence of any commercial or financial relationships that could be construed as a potential conflict of interest. The reviewers RR, XL and handling Editor declared their shared affiliation, and the handling Editor states that the process nevertheless met the standards of a fair and objective review.

## Footnotes

^1^http://nj.gov/health/eoh/rtkweb/documents/fs/1222.pdf
